# Complete Genome Sequences of Six *Listeria monocytogenes* Sequence Type 9 Isolates from Meat Processing Plants in Norway

**DOI:** 10.1128/genomeA.00016-18

**Published:** 2018-02-15

**Authors:** Annette Fagerlund, Solveig Langsrud, Birgitte Moen, Even Heir, Trond Møretrø

**Affiliations:** a Nofima, Norwegian Institute of Food, Fisheries and Aquaculture Research, Ås, Norway

## Abstract

*Listeria monocytogenes* is a foodborne pathogen that causes the often-fatal disease listeriosis. We present here the complete genome sequences of six *L. monocytogenes* isolates of sequence type 9 (ST9) collected from two different meat processing facilities in Norway. The genomes were assembled using Illumina and Nanopore sequencing data.

## GENOME ANNOUNCEMENT

*Listeria monocytogenes* is a Gram-positive opportunistic pathogen which causes outbreaks of foodborne disease. The main cause of listeriosis is the consumption of food contaminated from sources in food processing environments. In a previous study (1), we examined the diversity of *L. monocytogenes* strains in Norwegian meat and salmon processing plants, and a total of 680 *L. monocytogenes* strains were isolated and typed using multilocus variable-number tandem-repeat analysis (MLVA) (2). Three MLVA profiles, comprising 70% of the 313 strains isolated in the five sampled meat processing plants, were identified as multilocus sequence typing (MLST) sequence type 9 (ST9) (http://bigsdb.web.pasteur.fr/listeria/listeria.html) (3). Six of these strains, all isolated in 2012, were sequenced in the current study. Three were from plant M1, with strains MF4545 and MF4562 belonging to MLVA profile 16 and strain MF6172 with MLVA profile 21. MF4624, MF4626, and MF4697 were from plant M4 with MLVA profile 22.

For the purification of genomic DNA, cells were lysed using lysing matrix B and a FastPrep instrument (both MP Biomedicals), and DNA was isolated using the DNeasy blood and tissue kit (Qiagen), except for the MF4545 sample sequenced on the MinION instrument, for which cells were lysed using mutanolysin and DNA isolated using the Qiagen Genomic-tip 500/G kit.

For Illumina sequencing, Nextera XT libraries were prepared and sequenced either on a MiSeq platform with 300-bp paired-end (PE) reads (strains MF4545, MF4562, MF4624, and MF4697) or on a HiSeq platform with 150-bp PE reads (strains MF4626 and MF6172). For strains MF4626, MF4697, and MF6172, this was performed at the Norwegian Sequencing Centre (http://www.sequencing.uio.no). Raw Illumina reads were filtered at Q15 and trimmed of adaptors using fastq-mcf from the ea-utils package (4).

For sequencing using the Oxford Nanopore MinION instrument, sequencing libraries were prepared using the ligation sequencing kit 1D (product number SQK-LSK108) or, for strain MF6172, the 1D^2^ sequencing kit (SQK-LSK308). The libraries from strains MF4545, MF4624, MF4626, and MF4697 were barcoded using the Native barcoding kit (product number EXP-NBD103). For MF4545 and MF4562, library preps were prepared from unsheared DNA, and sequencing was performed on a FLO-MIN106 flow cell. For the remaining four genomes, the DNA was sheared to ∼8,000-kb fragments in a Covaris g-TUBE, and sequencing was performed on a FLO-MIN107 flow cell. Raw fast5 reads were base-called using ont-albacore version 1.2.2, and adapters were removed using Porechop version 0.2.1. For the reads from barcoded libraries, both ont-albacore and Porechop were run with barcode demultiplexing. The genomes were assembled using the Unicycler version 0.3.0b hybrid assembly pipeline (5), using Nanopore reads of > 1 kb. For MF6172, only 1D^2^ reads were used. Genomic features were identified and annotated using the NCBI Prokaryotic Annotation Pipeline (PGAP).

Genome sizes and read coverage data are listed in Table 1. All strains except MF4562 harbor a plasmid related to pLM58 from *L. monocytogenes* AT3E (6). The availability of these genome sequences will facilitate in-depth comparative analysis of the ST9 group of *L. monocytogenes* strains.

**TABLE 1:** Accession numbers and genome assembly characteristics for sequenced strains

Strain	Chromosome				Plasmid			
	GenBank accession no.	Length (bp)	Illumina coverage (×)	Nanopore coverage (×)	GenBank accession no.	Length (bp)	Illumina coverage (×)	Nanopore coverage (×)
MF4545	CP025443	3,010,323	211	17	CP025444	25,550	304	5
MF4562	CP025442	3,016,037	210	29				
MF4624	CP025259	3,030,702	200	203	CP025260	63,182	262	220
MF4626	CP025082	2,974,111	401	145	CP025083	58,524	647	225
MF4697	CP025438	3,030,421	306	134	CP025439	63,151	532	222
MF6172	CP025440	3,030,581	465	77	CP025441	63,182	652	111

### Accession number(s).

Nucleotide sequence accession numbers are listed in Table 1. The versions described in this paper are the first versions.
